# Chemodiversity of the Glucosinolate-Myrosinase System at the Single Cell Type Resolution

**DOI:** 10.3389/fpls.2019.00618

**Published:** 2019-05-21

**Authors:** Shweta Chhajed, Biswapriya B. Misra, Nathalia Tello, Sixue Chen

**Affiliations:** ^1^ Department of Biology, University of Florida, Gainesville, FL, United States; ^2^ Genetics Institute, University of Florida, Gainesville, FL, United States; ^3^ Section on Molecular Medicine, Department of Internal Medicine, Center for Precision Medicine, Wake Forest School of Medicine, Winston-Salem, NC, United States; ^4^ Plant Molecular and Cellular Biology, University of Florida, Gainesville, FL, United States; ^5^ Interdisciplinary Center for Biotechnology Research, University of Florida, Gainesville, FL, United States

**Keywords:** glucosinolate, myrosinase, cell type, metabolism, protein-protein interaction

## Abstract

Glucosinolates (GLSs) are a well-defined group of specialized metabolites, and like any other plant specialized metabolites, their presence does not directly affect the plant survival in terms of growth and development. However, specialized metabolites are essential to combat environmental stresses, such as pathogens and herbivores. GLSs naturally occur in many pungent plants in the order of Brassicales. To date, more than 200 different GLS structures have been characterized and their distribution differs from species to species. GLSs co-exist with classical and atypical myrosinases, which can hydrolyze GLS into an unstable aglycone thiohydroximate-O-sulfonate, which rearranges to produce different degradation products. GLSs, myrosinases, myrosinase interacting proteins, and GLS degradation products constitute the GLS-myrosinase (GM) system (“mustard oil bomb”). This review discusses the cellular and subcellular organization of the GM system, its chemodiversity, and functions in different cell types. Although there are many studies on the functions of GLSs and/or myrosinases at the tissue and whole plant levels, very few studies have focused on different single cell types. Single cell type studies will help to reveal specific functions that are missed at the tissue and organismal level. This review aims to highlight (1) recent progress in cellular and subcellular compartmentation of GLSs, myrosinases, and myrosinase interacting proteins; (2) molecular and biochemical diversity of GLSs and myrosinases; and (3) myrosinase interaction with its interacting proteins, and how it regulates the degradation of GLSs and thus the biological functions (e.g., plant defense against pathogens). Future prospects may include targeted approaches for engineering/breeding of plants and crops in the cell type-specific manner toward enhanced plant defense and nutrition.

## Introduction

One of the most extensively studied classes of anti-herbivore chemical defenses in plants is glucosinolates (GLSs), a group of sulfur-rich, amino acid-derived metabolites combining a β-d-glucopyranose residue linked *via* a sulfur atom to an *N*-hydroxyimino sulfate ester, which are plant-derived natural products (Halkier and Gershenzon, 2006; Halkier, 2016). GLSs are widely distributed in the order Brassicales, which includes vegetables (cabbage, cauliflower, and broccoli), spice plants supplying condiments (mustard, horseradish, and wasabi), and reference species, *Arabidopsis thaliana* (Fahey et al., 2001; Reichelt et al., 2002). Upon insect feeding or mechanical disruption, GLSs are hydrolyzed by myrosinases (thioglucoside glucohydrolase, TGG, EC 3.2.1.147) into unstable thiohydroximate-O-sulfonates, which rearrange to form different hydrolytic products such as isothiocyanates (ITCs), nitriles, and other by-products depending on the nature of the GLS side chain and the reaction conditions, such as iron, pH, and presence of myrosinase interacting proteins (Chen and Andreasson, 2001; Wittstock et al., 2016a). This GLS-myrosinase (GM) system is popularly known as “mustard oil bomb” (Lüthy and Matile, 1984; Ratzka et al., 2002). Myrosin cells (an idioblast cell type accumulating TGGs) are involved in plant defense by hydrolyzing GLSs into toxic volatiles such as ITCs or nitriles (Wittstock et al., 2003). TGGs are known to be present in all *A. thaliana* organs and were reported in *A. thaliana* and *B. napus* phloem parenchyma as well as in guard cells (Andréasson et al., 2001; Thangstad et al., 2004). In general, GLSs are enriched in “S-cells” that are found in *Arabidopsis* flower stalks and occur close to myrosin cells (Koroleva et al., 2000; Andréasson et al., 2001).

The spatial distribution of GLSs was demonstrated in *A. thaliana* leaves by constructing ion intensity maps from matrix-assisted laser desorption/ionization-time of flight (MALDI-TOF) mass spectra, where major GLSs were found to be more abundant in tissues of the midvein and the periphery of the leaf than the inner lamina (Shroff et al., 2008). Although this study concluded that GLSs are not abundant on *A. thaliana* leaf surfaces, the authors could not obtain information on the cell type distribution of GLSs in leaves. Moreover, all the genes in the GLS biosynthetic pathways have been identified, and it is somewhat known where GLSs are stored (Koroleva et al., 2000; Andréasson et al., 2001), but it has remained elusive where GLSs are specifically produced at the subcellular, cellular, and tissue levels (Rask et al., 2000; Nintemann et al., 2017). Neither is it clear about the cellular and subcellular compartmentation of different myrosinases and their interacting proteins, which include myrosinase-binding proteins (MBPs), myrosinase-associated proteins (MyAPs), and different specifier proteins.

In the following sections, we discuss various aspects of the GM system based on current knowledge, starting from the cellular control of enzymes, cell type, and subcellular organization, to uniqueness of myrosinases and myrosinase interacting proteins covering a range of small molecule and macromolecular interactions of the “mustard oil bomb.”

## The Glucosinolate-Myrosinase System and Cellular Control of Enzyme Reactions

**Figure 1:**
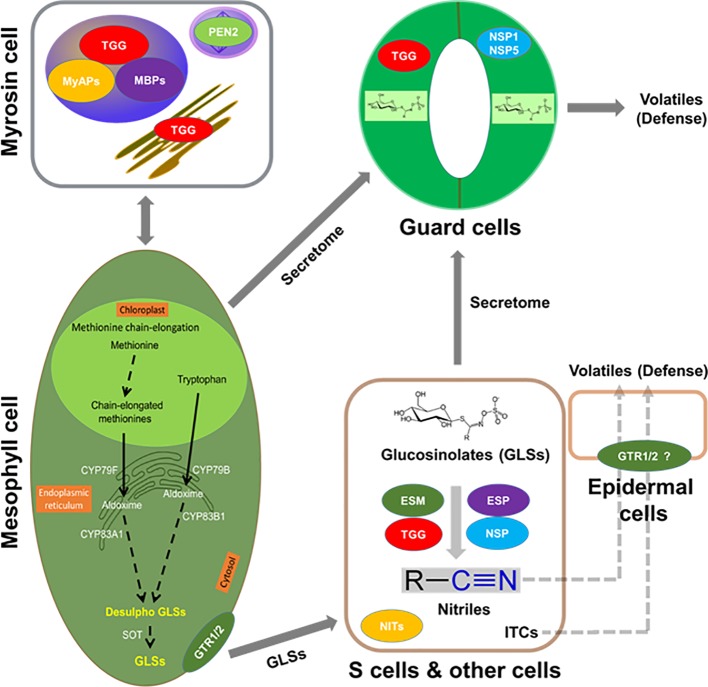
Putative interactions between myrosinases (TGGs), myrosinase interacting proteins, GLSs, and volatiles in the context of cell type compartmentation. A myrosin cell shows vacuolar localization of TGGs, myrosinase-binding proteins (MBPs), and myrosinase-associated proteins (MyAPs); peroxisomal localization of penetration (PEN2); and ER localization of TGG. Transporters that are specific to GLSs such as NRT1/PTR glucosinolate transporter (GTR) 1, GTR2 or non-specific transporters could be aiding in their transport to site of accumulation such as S-cells or guard cells. Importantly, these cells may have the capability of *de novo* biosynthesis of GLSs. In addition, the presence of epithiospecifier modifier (ESM, MyAP-like), epithiospecifier (ESP), and nitrile specifier (NSP) 1, NSP5, etc. may lead to the breakdown of GLSs to nitriles and isothiocyanates (ITCs) for roles in cell type-specific signaling and defense against pathogen and herbivores. GLSs, TGGs, and ESP were found in the S-cells, and the presence of ESM and NSP is indicative of other cell types.

As found in the order of Brassicales, including important crops (e.g., mustard, oilseed rape, radish, broccoli, and cabbage), GLSs co-exist with myrosinases. When tissue damage occurs, the “mustard oil bomb” is detonated and GLSs are hydrolyzed and converted to different degradation products with a variety of biological activities (Rask et al., 2000; Halkier and Gershenzon, 2006; Yan and Chen, 2007; Bednarek et al., 2009; Clay et al., 2009; Halkier, 2016; Wittstock et al., 2016a). For example, these degradation products play important roles in plant defense against pathogens and herbivores, as well as serve as attractants to specialists (Rask et al., 2000; Barth and Jander, 2006; Clay et al., 2009; Wittstock et al., 2016a). Several of these degradation products are involved in plant nutrition (Holmes, 1980; Armengaud et al., 2004) and growth regulation (Hasegawa et al., 2000; Hull et al., 2000; Mikkelsen et al., 2000). In plant metabolism, it is important that enzymes and substrates are under tight regulation, which is more relevant for toxic compounds, as these chemical defenses are derived from specialized metabolites. There are several ways of regulation: (1) coarse control through biosynthesis; (2) fine control of enzyme activity through protein interaction and allosteric regulation; and (3) substrate and enzyme compartmentalization (Sweetlove and Fernie, 2013). While the regulation is well studied in primary metabolism (e.g., photosynthesis and respiration), it is not clear in many of the specialized metabolic processes such as GLS metabolism. Furthermore, protein-protein interactions are intrinsic to virtually every cellular process and have been extensively studied in animals and yeast (Uetz et al., 2000; Gavin et al., 2002; Ho et al., 2002; Li et al., 2004; Huttlin et al., 2017). In plants, this area has lagged behind in spite of recent progress (Hosseinpour et al., 2012; Zhang et al., 2016; Jiang et al., 2018). Vast majority of the studies did not go beyond identifying physical interactions to the point of functional analysis. Figure 1 shows the potential molecular interactions of the GM system in the context of cell type-specific metabolisms.

## Cell Type-Specific Cellular and Subcellular Organization of the “Mustard Oil Bomb”

Myrosinase is located in myrosin cells, which are scattered cells in radicles, stems, leaves, petioles, seeds, and seedlings of several species (Husebye et al., 2002). A cell-specific localization was found in radicles and cotyledons of the maturing embryo resembling the pattern of the myrosin cells (Bones et al., 1991). Most GLSs are constitutively present in all *Arabidopsis* tissues (Petersen et al., 2002; Brown et al., 2003). The key steps in the biosynthesis of the different types of GLSs are localized in distinct cells in separate as well as overlapping vascular tissues (Nintemann et al., 2018). The presence of GLS biosynthetic enzymes in parenchyma cells of the vasculature may assign new defense-related functions to these cell types (Nintemann et al., 2018). To date, the cellular and subcellular compartmentation of the “mustard oil bomb” (Lüthy and Matile, 1984) is not completely clear and is rather contradictory. For instance, in *Arabidopsis* flower stalks, GLSs were found in the elongated sulfur-rich “S-cells” situated between phloem and endodermis (Koroleva et al., 2000; Husebye et al., 2002). However, the myrosinase TGG1 was found to be abundant in guard cells, whereas TGG1 and TGG2 were localized to the phloem-associated cells close to the “S-cells” (Husebye et al., 2002; Thangstad et al., 2004; Barth and Jander, 2006). Thus, it appears that myrosinases and their substrates were physically separated in the plant tissues. However, such an arrangement may not be the case as a recent proteomics study located the myrosinases in “S-cells” (Koroleva and Cramer, 2011). In *Brassica juncea* seedlings, myrosinase was found to co-localize with GLSs in aleurone-type cells (Kelly et al., 1998). In *Arabidopsis* suspension cells, both myrosinases and GLSs were present (Alvarez et al., 2008). Such diverse co-localization results may indicate that myrosinases and GLSs are spatially separated at the subcellular levels. Alternatively, they could be in the same compartment with tight control of myrosinase activities. GLSs were found in vacuoles rich in ascorbic acid (Grob and Matile, 1979), which plays a role to inhibit myrosinase at high concentration and activate myrosinase at low concentration. This dual regulation supports the potential co-localization of GLSs and myrosinases in the same subcellular compartment.

Recent metabolomics data have confirmed the presence of GLSs in guard cells (Geng et al., 2016; Zhu and Assmann, 2017) and revealed the changes in GLS metabolism in guard cells upon treatment with CO_2_ (Geng et al., 2016) and ABA (Zhu and Assmann, 2017). The first indication of the role of GLS metabolism in stomatal movement was obtained through analysis of the effect of ABA on stomatal movement of the *Arabidopsis* myrosinase mutant *tgg1* (Zhao et al., 2008). Subsequently, additional reverse genetics studies corroborated the role of GLS metabolism in stomatal movement (Islam et al., 2009; Zhu et al., 2014). Furthermore, stomatal closure was induced by pharmacological treatments with different GLS hydrolysis products (Khokon et al., 2011; Sobahan et al., 2015). However, these products and the amounts used are of synthetic origin and abundance. It is not known what degradation products are produced and how much *in vivo*, which GLSs and myrosinases [TGGs and/or Penetration 2 (PEN2)] are involved, and how protein interactions regulate the GLS breakdown in guard cells.

**Figure 2:**
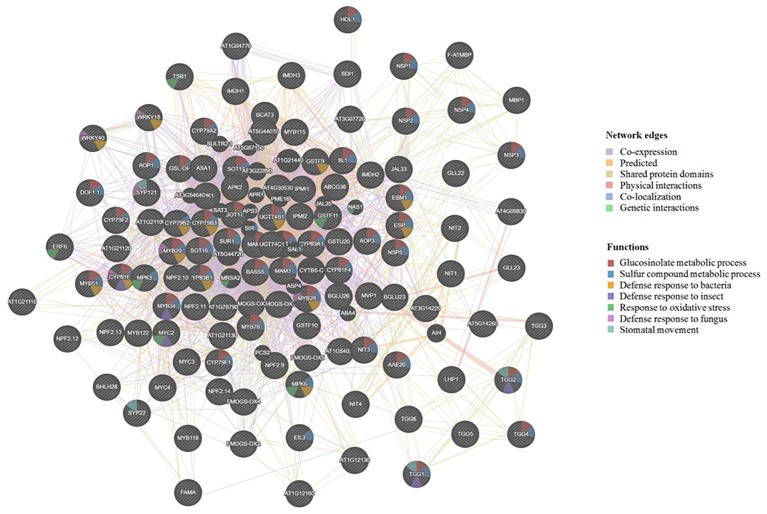
Visualization of functional prediction of protein networks in the glucosinolate-myrosinase (GM) system using GeneMANIA (http://genemania.org/). The protein names are indicated inside the nodes, and the links between the nodes indicate the network edges in which the proteins are connected. The color of the edges represents evidence for the connection, which includes co-expression (purple), predicted (yellow), shared protein domains (beige), physical interactions (pink), co-localization (blue), and genetic interactions (green). As to functions associated with each protein, the color code inside the nodes indicates GLS metabolic process (red), sulfur compound metabolic process (blue), defense response to bacterium (yellow), defense response to insect (purple), response to oxidative stress (green), defense response to fungus (pink), and stomatal movement (light blue).

The *Arabidopsis cyp79b2*/*cyp79b3* mutants are known to produce mostly aliphatic GLSs (Zhao et al., 2002; Chen et al., 2003; Grubb and Abel, 2006; Khokon et al., 2011; Sobahan et al., 2015), while the *myb28/myb29* mutants are known to produce mostly indolic GLSs (Hirai et al., 2007; Beekwilder et al., 2008). Furthermore, the *tgg1/tgg2* double mutant showed undetectable myrosinase activity, and damage-induced breakdown of endogenous GLSs was not from aliphatic GLSs and was greatly slowed for indole GLSs (Barth and Jander, 2006). Moreover, the *tgg1/tgg2* mutant lacking the foliar myrosinases was compromised in activation of their GLS defense. Another mutant, *atvam3* mutant showed abnormal distribution of myrosin cells and overproduction of TGG1 and TGG2 (Ueda et al., 2006). Thus, beyond TGGs, MYB28, MYB29, AtVAM, CYP79s, and other biosynthetic genes all affect GLS deposition levels and possibly cell type specificity of the GM system. To understand the regulation and correlation of these proteins, we used GeneMANIA software (Warde-Farley et al., 2010) and predicted the association of the known genes involved in GLS metabolism (from our selected gene list in Supplementary Table S1). This software further added putative proteins with similar functions and potential involvement in the GM system (Figure 2).

In *B. napus* leaves, myrosinases are localized in mesophyll cells and phloem cells (Chen and Andreasson, 2001) and were mainly stored in protein-rich vacuolar structures of myrosin cells (Rask et al., 2000; Ueda et al., 2006). There is also a report of the presence of myrosinase as cytosolic enzymes bound to intracellular membranes (Lüthy and Matile, 1984). The knowledge of the localization of myrosinases and interacting proteins was advanced by vacuolar proteomics. Myrosinases, TGG1 and TGG2, and myrosinase-associated protein (MyAP) 1 were identified in the vacuoles. In the early leaf developmental stages, TGG1 is more abundant than TGG2, whereas in fully expanded leaves, both TGG1 and TGG2 levels show increased accumulation. Concurrently, MyAP1 levels are increasingly abundant. We have previously observed such regulation of myrosinase expression, which correlated with GLS turnover (Petersen et al., 2002). The co-localization of myrosinase and MyAP1 and the concurrent expression during development lead to the hypothesis that the vacuolar myrosinases may be active and MyAPs may interact with myrosinase to play a role in GLS hydrolysis. For example, MyAPs may facilitate ITC production (Zhang et al., 2006). Indeed, immunogold analysis of leaf sections showed the presence of TGG1 and TGG2 in the same vacuoles (Ueda et al., 2006). An independent vacuolar proteomics study also identified these proteins (Carter et al., 2004). In addition, two more MyAPs (At1g54000 and At1g54010) and three myrosinase-binding proteins (MBPs) (At1g52040, At3g16470, and At2g39330) were localized in the vacuoles (Carter et al., 2004). TGG1 and TGG2 were also found in the endoplasmic reticulum (ER), ER bodies, and transvacuolar strands, and this localization is dependent on MyAP1 (MVP1). Mutation of the MyAP1 clearly altered the subcellular localization profiles of the green fluorescent protein (GFP)-tagged TGG1 and TGG2 (Agee et al., 2010). Interestingly, the myrosinase PEN2 (hydrolyzing indole GLSs and shown to function in plant defense (Bednarek et al., 2009; Clay et al., 2009; Millet et al., 2010; Fan et al., 2011; Johansson et al., 2014; Frerigmann et al., 2016; Luti et al., 2016; Xu et al., 2016; Vilakazi et al., 2017)) is targeted to peroxisomes and the outer mitochondrial membrane (Fuchs et al., 2016). In addition to MyAPs and MBPs, specifier proteins including epithiospecifier modifier (ESM, MyAP-like), epithiospecifier protein (ESP), nitrile specifier protein (NSP), and thiocyanate forming protein (TFP) may affect the outcome of GLS degradation (Lambrix et al., 2001; Burow et al., 2006; Zhang et al., 2006; Wittstock et al., 2016a,b ; Backenköhler et al., 2018). ESP was found to be in “S-cells” and in guard cells with NSP1 and NSP5 (Burow et al., 2007; Zhao et al., 2008). The functions of these MyAPs, MBPs, and specifier proteins in “S-cells” and guard cells and their interactions with myrosinases in different cell types are not known.

**Figure 3:**
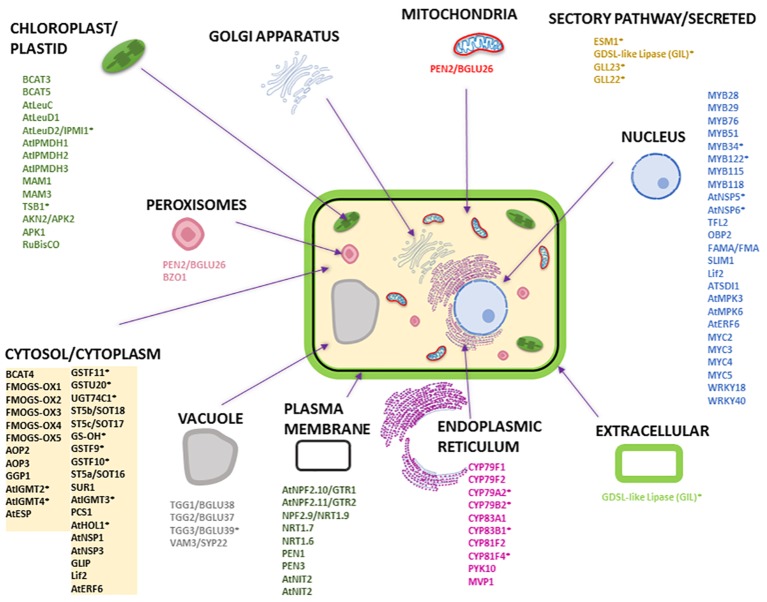
Subcellular localization of *Arabidopsis* proteins involved in GLS biosynthesis, degradation, and transport. The subcellular organelles and their localized proteins are given in similar color. Most of the subcellular localizations are based on literature evidence. The names of the proteins with an asterisk are predicted localization information (i.e., for proteins that do not have localization information in literature) that are based on consensus from at least three software tools used. The prediction tools used were Eplant, TargetP, LocTree, ngLOC, Plant-mPLoc, and TAIR. Please refer to Supplementary Table S1 for detailed information.

To understand the subcellular organization of the GM system, we compared the proteins and pathways involved in GLS biosynthesis, degradation, and transport using available and/or predicted subcellular localization information. Figure 3 and Supplementary Table S1 provide an overview of the GM system at subcellular level based on available literature and analysis using different protein localization tools: (1) Plant-mPLoc^1^ (Chou and Shen, 2007, 2008, 2010); (2) TAIR^2^ (with annotation based on literature); (3) Eplant^3^ using SUBA (Subcellular Localisation Database for *Arabidopsis*) with annotation based on subcellular proteomics and/or protein fluorescence microscopy; (4) TargetP^4^ based on the N-terminal targeting sequences (chloroplast transit peptide (cTP), mitochondrial targeting peptide (mTP), or secretory pathway signal peptide (SP) (Emanuelsson et al., 2000) [with a reliability score of 1–5 (1 being most reliable and 5 least reliable)]; (5) LocTree^5^ using support vector machines for localization prediction (in the form of expected accuracy); and (6) ngLOC^6^ using Bayesian method for prediction of localization. As shown in Figure 3, most GM system proteins were found to be in the cytoplasm followed by nucleus, where the transcriptional regulators were localized. All the cytochrome P450s involved in GLS biosynthesis and modification were localized to endoplasmic reticulum, and other GLS biosynthesis-related proteins were in the chloroplast and cytoplasm. Glucosinolate transporters (GTR1 and GTR2) and nitrate transporters (NRT1.6, NRT1.7, and NRT1.9) were found to be in plasma membrane. It is not known how glucosinolates are transported into vacuoles. PEN2 and BZO1 were localized in peroxisomes. No GM system proteins were found on Golgi apparatus. Out of the 114 GM system proteins used in this study (Supplementary Table S1), 65 proteins had experimental evidence of localization, 26 were predicted using the software tools (at least three tools with consistent result), and 23 proteins could not be conclusively localized.

## Distinct Molecular and Biochemical Properties of Myrosinases

Myrosinases are classified into two types, typical (classical) and atypical myrosinases. The crystal structure of a classical myrosinase shows that the protein folds into an (*β*/*α*)_8_ barrel structure (Burmeister et al., 1997). In the active site, a Glu (E) residue is involved in nucleophilic attack to initiate the release of an aglycone (thiohydroximate-O-sulfonate) and form a glucosyl-enzyme intermediate. Another Gln (Q) residue enables the hydrolysis of this intermediate with assistance from water and ascorbate. Classical myrosinases (with QE catalytic residues) use ascorbate as a cofactor and proton donor to facilitate the release of bound glucose (Burmeister et al., 1997; Wittstock and Burow, 2010; Bhat and Vyas, 2019). In contrast, atypical myrosinases have two catalytic Glu residues (EE), which function as acid/base catalyst in the active site. They do not require ascorbate. In addition, atypical myrosinases have two basic amino acid residues at different positions (+6 and +7) for glucosinolate binding compared to +0 position arginine residue of classical myrosinases (Wittstock and Burow, 2010; Nakano et al., 2017; Shirakawa and Hara-Nishimura, 2018; Bhat and Vyas, 2019). Classical myrosinases are glycosylated, activated by low concentrations of ascorbate, and accepted GLSs as the only substrates (Chen and Halkier, 1999; Chen and Andreasson, 2001). In contrast, atypical myrosinases, such as PEN2 and PYK10, can hydrolyze indole GLSs and also use O-glucosides as substrates (Bednarek et al., 2009; Nakano et al., 2017). Although myrosinase does not use acylated GLSs and desulpho-GLSs as substrates, it may accept a wide range of GLS substrates (Chen and Halkier, 1999; Rask et al., 2000; Barth and Jander, 2006). Myrosinases from *B. napus* and *Crambe abyssinica* degrade different GLS at different rates (James and Rossiter, 1991; Finiguerra et al., 2001). However, the mechanism underlying this substrate specificity is not established. Myrosinases in *B. napus* are encoded by >29 genes in three subfamilies, denoted as MA, MB, and MC. The MA myrosinases occur as dimers, while MB and MC myrosinases exist in complexes with MBPs and/or MyAPs (Lenman et al., 1990; Rask et al., 2000). By heterologous expression in yeast, we have previously produced a functional free form myrosinase Myr1 from the MB subfamily (Chen and Halkier, 1999). The activity of this Myr1 suggests that MBPs and MyAPs are not absolutely necessary for myrosinase activity, but raises questions on the functions of MBPs and MyAPs and their interactions with myrosinases.

Bioinformatic analysis of the *Arabidopsis* genome revealed the presence of six myrosinase genes *TGG1*-*TGG6* (Xu et al., 2004). *TGG1* and *TGG2* are expressed in leaves (Xue et al., 1995; Husebye et al., 2002; Thangstad et al., 2004; Barth and Jander, 2006; Ueda et al., 2006) and flowers (Ruan et al., 1998; Barth and Jander, 2006), while *TGG4* and *TGG5* are specifically expressed in roots (Zimmermann et al., 2004). *TGG3* and *TGG6* are pseudogenes (Husebye et al., 2002; Zhang et al., 2002). Although TGG1 and TGG2 appear to display a low degree of substrate specificity, the activities of TGG1 and TGG2 have been correlated with the feeding preference and growth of different generalist and specialist insects (Barth and Jander, 2006). Interestingly, overexpression of TGG1 and TGG2 leads to accumulation of several GLS degradation products, including 5-methylhexanenitrile, heptanenitrile, 1-isothiocyanato-3-methylbutane, 1-isothiocyanato-4-methylpentane, and 1-isothiocyanato-3-methylhexane. Based on the degradation product profile, possible endogenous substrates for the two TGGs include 4-methylthiobutylglucosinolate, 4-methylpentylglucosinolate and 3-methylbutylglucosinolates (Ueda et al., 2006). Investigating endogenous substrates of different classical and atypical myrosinases is an important future direction.

In leaves, TGG1 was found to be abundant in guard cells, while TGG2 appeared only present in phloem-associated cells (Barth and Jander, 2006; Zhao et al., 2008). Considering the presence of GLSs in guard cells (Geng et al., 2016; Zhu and Assmann, 2017), how the GM system plays a role in guard cell functions (e.g., stomatal immunity) is an interesting question. Clearly, mutation of the TGG1 and/or TGG2 genes affected the guard cell size, stomatal aperture, and leaf metabolites, such as fatty acids, glucosinolates, and indole compounds (Ahuja et al., 2016). Another proteomic study of trichome and epidermal pavement cells did not identify the TGG1 protein in the samples (Wienkoop et al., 2004). However, a single cell type study in trichomes found the presence of gene encoding transcription factors of aliphatic GLS (MYB28, MYB29 and MYB76) and indole GLS (MYB34, MYB51 and MYB122), indicating that trichomes have biosynthetic genes for the GM system (Frerigmann et al., 2012), but nothing was suggested about myrosinases activity or expression. Given the defense roles of guard cells and trichomes, characterization of the GM systems in these special cell types is of great importance to understand the molecular mechanisms underlying the cell type-specific functions, e.g., defense against pathogen invasion.

## Complex Formation Between Myrosinase and Its Interacting Proteins

As described earlier, myrosinase interacting proteins include MBP, MyAP, and specifier proteins (ESM, ESP, NSP, and TFP). The first six MBPs identified in *B. napus* range in size from 30 to 110 kDa (Taipalensuu et al., 1996; Chisholm et al., 2000). All MBPs contain jacalin-like repeats (Chisholm et al., 2000; Andréasson et al., 2001). Jacalin-related proteins share the domain structure of plant lectins and are upregulated by phytohormones (e.g., jasmonic acid, salicylic acid, and ethylene) and pathogens (Taipalensuu et al., 1996; Geshi and Brandt, 1998; Xiang et al., 2011; Vilakazi et al., 2017). Recently, identification of bacterial lipopolysaccharide interacting proteins in *Arabidopsis* revealed myrosinases, TGG1 and TGG2, and a MBP (Vilakazi et al., 2017). It remains unclear how the MBP levels are regulated and whether MBPs directly interact and affect myrosinase activity and specificity. In *B. napus* seeds, MBPs are present in most cells but not in the myrosin cells (Rask et al., 2000; Ueda et al., 2006). During germination, MBPs are co-localized with myrosinases in cotyledons, suggesting that preformed myrosinase complexes do exist (Geshi and Brandt, 1998; Eriksson et al., 2002). Using basic local alignment search tool (BLAST) to interrogate the *Arabidopsis* genome reveals >30 putative MBPs. MBP1 and MBP2 are like lectin jacalins and plant aggregating factors. MBP1 and MBP2 are abundantly expressed in immature flowers, and the pattern is similar to that of myrosinase TGG1 (Capella et al., 2001). MBP expression and myrosinase activity are affected in the *coi1* mutant, which is insensitive to jasmonate (Capella et al., 2001). Depletion of MBPs does not alter the cellular distribution of myrosinases but prevents myrosinases from forming complexes (Eriksson et al., 2002). Thus, the functions of MBPs are not fully understood. Interestingly, most NSPs possess jacalin-like domains and are MBP-like (Kuchernig et al., 2012). The jacalin-like domain may interact with the glycans of myrosinases to potentially affect GLS degradation. However, experimental evidence is lacking. NSPs were shown to enhance simple nitrile formation (He et al., 2009; Kissen and Bones, 2009; Chen et al., 2015; Wittstock et al., 2016b). Recently, iron was shown to be a centrally bound cofactor of ESP, TFP, and NSP involved in glucosinolate breakdown. In addition, NSP active site has fewer restrictions to the aglycone conformation than ESP and TFP. This may explain why NSP facilitates simple nitrile production, but not production of epithionitrile and thiocyanate that may need exact positioning of the aglycone thiolate relative to the side chain (Backenköhler et al., 2018). In addition to MBPs, MyAPs form complexes with myrosinases in *B. napus* (Taipalensuu et al., 1996). In *Arabidopsis*, TGG2 was pulled down with MyAP1 in leaf extracts (Agee et al., 2010). MyAPs display high similarity to GDSL lipases, which have a motif of Gly, Asp, Ser, and Leu residues in the active site. The *Arabidopsis* genome contains >80 genes encoding GDSL lipases, typically with a GDSL-like motif, a catalytic triad of Ser, Asp and His residues, and a lipase signature sequence GxSxxxxG (Brick et al., 1995). The possible lipase activity of MyAP suggests a potential role of MyAP in releasing acyl groups from acylated GLSs, thereby making them available for myrosinase hydrolysis. *Arabidopsis* contains acylated GLSs in seeds, but *B. napus* does not contain acylated GLSs; thus, MyAP in *B. napus* may have other functions. A recent study shows that overexpression of *B. napus MyAP1* led to enhanced plant defense against a fungal pathogen *Sclerotinia sclerotiorum* (Wu et al., 2017). A MyAP-like ESM was found to favor ITC production and protect *Arabidopsis* from herbivory (Zhang et al., 2006). However, whether this system involves myrosinase complex formation is still not known. In some plants, ESPs are involved in GLS hydrolysis (Foo et al., 2000; Burow et al., 2006). Hydrolysis of alkenyl GLSs in the presence of ESP leads to the formation of nitriles or epithionitriles, instead of isothiocyanates (Zabala Mde et al., 2005; Burow et al., 2006). Because ESPs can alter the course of hydrolysis, they are important in determining plant herbivore choice and host resistance (Lambrix et al., 2001). Furthermore, this suggests that ESP is situated close to the active site so that it could promptly convert the unstable aglycone to nitriles. Although kinetic studies have showed that ESP acts as a non-competitive inhibitor of myrosinase (MacLeod and Rossiter, 1985), no stable interaction between ESP and myrosinase has been reported (Burow et al., 2006). Like nitrile formation, the production of thiocyanate was found to be associated with TFP. For detailed description of myrosinase specifier proteins, please refer to a recent review (Wittstock et al., 2016a). In summary, several other groups of proteins may interact with myrosinases and function to affect how GLSs are degraded, leading to the formation of different metabolic products. Systematic studies to characterize the interaction of these proteins with myrosinases are needed to elucidate their specific functions.

## Directions for Future Research and Conclusions

Currently, the cellular and subcellular location of myrosinases, GLSs, and their interacting proteins, i.e., the GM system, are far from established. Given >100 cell types in plants and >5,500 species of GLS producers, it would be a challenge to capture all the species-specific and cell type-specific information of the “mustard oil bomb.” In addition, with temporal accumulation and expression patterns of metabolites and enzymes involved typically in case of specialized metabolites, these eventual pictures could be very complex. Using cell type-specific genetic manipulations (e.g., GFP fusion and CRISPR), one can envision to capture the cell type-specific expression patterns and functional role of the glucosinolate biosynthetic proteins, myrosinases, and myrosinase interacting proteins. There exist large gaps in the knowledge base of the GM system, e.g., myrosinase interacting proteins in terms of their interactions, co-localizations, regulations, and functions in specific cell types. Furthermore, the developmental staged appearance and regulation of the proteins and metabolites are not clear. Moreover, the reported interactions of myrosinases and other proteins could be very much cell type-specific or subcellular localized, which is not well studied till date. Without resolving the cell type specificity of the proteins and metabolites, it would be very challenging to draw mechanistic conclusions on the specific roles of the enzymes, interactors, transporters, and the metabolites from tissue- and whole plant-based data where the information are averaged out (Dai and Chen, 2012; Misra et al., 2014).

In the future, efforts need to focus on large-scale speedy preparations of organelles and subcellular fractions (e.g., vacuoles, peroxisomes, and chloroplasts) in a time-dependent manner to capture the dynamics of protein interactions and GLS metabolism. It is obviously challenging to prepare and enrich plant cell types (e.g., the “S-cells”) in copious amounts for more system-wide experiments such as transcriptomics, proteomics, and metabolomics and to obtain preparations at a given time and for a specific treatment. With the recent development of single-cell omics tools (Misra et al., 2014; Efroni and Birnbaum, 2016; Doerr, 2019), such large-scale molecular characterization of different single cells is within sight and will greatly enhance the understanding of the chemodiversity of the GM system at the single-cell resolution.

## Author Contributions

SChh made the list of protein localization, Figures 2, 3, generated the reference list, and edited the manuscript. BM wrote one-third of the manuscript draft, made Figure 1 draft, and edited the manuscript. NT assisted with Supplementary Table S1 analysis, reference list, and edited the manuscript. SChe designed the manuscript, wrote two-third of the text, provided guidance to students, and finalized the manuscript for submission.

### Conflict of Interest Statement

The authors declare that the research was conducted in the absence of any commercial or financial relationships that could be construed as a potential conflict of interest.

## Footnotes

^1^http://www.csbio.sjtu.edu.cn/bioinf/plant-multi/

^2^https://www.arabidopsis.org/tools/bulk/protein/index.jsp

^3^https://bar.utoronto.ca/eplant/

^4^http://www.cbs.dtu.dk/services/TargetP/

^5^https://rostlab.org/services/loctree3/

^6^http://genome.unmc.edu/ngLOC/index.html
