# Supplementary material for: Chemodiversity of the Glucosinolate-Myrosinase System at the Single Cell Type Resolution
Source: Front Plant Sci. 2019 May 21;10:618. doi: 10.3389/fpls.2019.00618 (PMC6536577; doi:10.3389/fpls.2019.00618)
Supplement: Supplementary file 1 [file Table_1.pdf]

Supplemental Table 1. Subcellular localization of Arabidopsis proteins involved in glucosinolate-myrosinase (GM) system using six different tools (Plant-mPLoc, TAIR, Eplant, TargetP, LocTree and ngLOC) along with evidence from literature. The six software tools are: a) Plant-mPLoc (http://www.csbio.sjtu.edu.cn/bioinf/plant-multi/); (b) TAIR (https://www.arabidopsis.org/tools/bulk/protein/index.jsp); (c) Eplant (https://bar.utoronto.ca/eplant/); (d) TargetP (http://www.cbs.dtu.dk/services/TargetP/). Scores are from 1 to 5, with 1 indicating the strongest prediction; (e) LocTree (https://rostlab.org/services/loctree3/); (f) ngLOC (http://genome.unmc.edu/ngLOC/index.html). LocTree and ngLOC use a 0 to 100 scale, with 100 being the most probable prediction. The final score indicates out of the six tools the number of consensus predictions. Ribulose-1,5-bisphosphate carboxylase/oxygenase (RuBisCO) was used as a positive control for subcellular localization prediction.

Gene name	Locus	Abbreviation	Swiss Prot ID	Amino acid	Plant-mPLoc[a]	TAIR[b]	Eplant[c]/Score		TargetP[d]/Score		LocTree[e]/Score		ngLOC[f]/Score		Final score/Localization		References
**ALIPHATIC GLUCOSINOLATE BIOSYNTHESIS AND MODIFICATION**																	
Branched-chain amino acid aminotransferase 3	AT3G49680	BCAT3	Q9M401	413	Chloroplast	Chloroplast	Plastid	88	Chloroplast	2	Chloroplast	99	Chloroplast	74	5	Chloroplast	Diebold et al., 2002
Branched-chain amino acid aminotransferase 4	AT3G19710	BCAT4	Q9LE06	354	Chloroplast	Undefined	Cytosol	54	Any other	3	Cytoplasm	99	Cytoplasm	27	2	Cytosol	Schuster et al., 2006
Bile acid transporter 5	AT4G12030	BAT5	Q94A17	407	Cell membrane	Other (e.g. Cytoplasm)	Plastid	22	Chloroplast	4	Golgi membrane	86	Plasma membrane	15	2	Chloroplast	Diebold et al., 2002
Isopropylmalate isomerase large subunit 1	AT4G13430	AtLeuC	Q94AR8	509	Chloroplast, Mitochondrion	Chloroplast	Plastid	58	Chloroplast	1	Mitochondrion	81	Nucleus	13	3	Chloroplast	He et al., 2010
Isopropylmalate isomerase small subunit 1	AT2G43100	AtLeuD1	Q9ZW84	256	Cytoplasm, Mitochondrion	Chloroplast	Plastid	56	Chloroplast	1	Mitochondrion	87	Chloroplast	16	3	Chloroplast	He et al., 2010; Knill et al., 2009
Isopropylmalate isomerase small subunit 2	AT3G58990	AtLeuD2	Q9LYT7	253	Mitochondrion	Chloroplast	Plastid	46	Chloroplast	1	Cytoplasm	86	Chloroplast	16	3	Chloroplast	
Isopropylmalate dehydrogenase 1	AT5G14200	AtIPMDH1	Q9FMT1	409	Chloroplast, Cytoplasm	Undefined	Plastid	64	Any other	5	Chloroplast	96	Chloroplast	65	3	Chloroplast	He et al., 2009
Isopropylmalate dehydrogenase 2	AT1G80560	AtIPMDH2	P93832	405	Chloroplast, Cytoplasm	Undefined	Plastid	70	Chloroplast	4	Chloroplast	97	Chloroplast	68	4	Chloroplast	He et al., 2010
Isopropylmalate dehydrogenase 3	AT1G31180	AtIPMDH3	Q9SA14	404	Chloroplast, Cytoplasm	Undefined	Plastid	64	Mitochondrion	5	Chloroplast	96	Chloroplast	65	3	Chloroplast	He et al., 2010
Methylthioalkylmalate synthase 1	AT5G23010	MAM1	Q9FG67	506	Chloroplast	Chloroplast	Plastid	70	Chloroplast	1	Chloroplast	95	Chloroplast	53	5	Chloroplast	Kroymann et al., 2001
Methylthioalkylmalate synthase 3	AT5G23020	MAM3	Q9FN52	503	Chloroplast	Chloroplast	Plastid	56	Chloroplast	3	Chloroplast	99	Chloroplast	77	5	Chloroplast	Textor et al., 2007
Cytochrome P450 79F1	AT1G16410	CYP79F1	Q949U1	538	Endoplasmic reticulum (ER)	Secreted	ER	46	Secretory	1	ER	99	ER	82	4	ER	Reintanz et al., 2001
Cytochrome P450 79F2	AT1G16400	CYP79F2	Q9FUY7	537	ER	Secreted	ER	48	Secretory	1	ER	99	ER	81	4	ER	Reintanz et al., 2001
Cytochrome P450 83A1	AT4G13770	CYP83A1	P48421	502	ER	Undefined	ER	38	Secretory	5	ER	84	ER	12	4	ER	Nintemann et al., 2017
Glutathione S-transferase F11	AT3G03190	GSTF11	Q96324	214	Cytoplasm	Undefined	Cytosol	24	Any other	5	Cytoplasm	93	Cytoplasm	23	3	Cytoplasm	
Glutathione S-transferase TAU 20	AT1G78370	GSTU20	Q8L7C9	217	Cytoplasm	Undefined	Cytosol	24	Mitochondrion	4	Cytoplasm	95	Cytoplasm, Nucleus	47	3	Cytoplasm	
UDP-glucosyl transferase 74C1	AT2G31790	UGT74C1	Q9SKC1	457	Cell membrane	Undefined	Mitochondrion	18	Any other	3	Cytoplasm	84	Cytoplasm	14	2	Cytoplasm	
Sulfotransferase 5b	AT1G74090	ST5b/AtSOT18	Q9C9C9	350	Cytoplasm	Other (e.g., Cytoplasm)	Cytosol	38	Any other	2	Cytoplasm	99	Cytoplasm	82	3	Cytoplasm	Klein et al., 2006
Sulfotransferase 5c	AT1G18590	ST5c/AtSOT17	Q9FZ80	346	Cytoplasm	Other (e.g., Cytoplasm)	Cytosol	34	Any other	2	Cytoplasm	99	Cytoplasm	82	3	Cytoplasm	Klein et al., 2006
Flavin-monooxygenase glucosinolate S-oxygenase 1	AT1G65860	FMOGS-OX1	Q9SS04	459	Chloroplast, Cytoplasm	Undefined	Cytosol	20	Any other	5	Chloroplast	88	Plasma membrane	13	2	Cytosol	Li et al., 2010
Flavin-monooxygenase glucosinolate S-oxygenase 2	AT1G62540	FMOGS-OX2	Q94K43	457	Chloroplast	Undefined	Cytosol	10	Secretory	5	Cytoplasm	87	Chloroplast	12	2	Cytosol	Li et al., 2010
Flavin-monooxygenase glucosinolate S-oxygenase 3	AT1G62560	FMOGS-OX3	Q9SXE1	462	Chloroplast	Undefined	Plastid	12	Chloroplast	5	Chloroplast	89	Chloroplast	13	4	Cytosol	Li et al., 2010
Flavin-monooxygenase glucosinolate S-oxygenase 4	AT1G62570	FMOGS-OX4	Q93Y23	461	Chloroplast	Undefined	Cytosol	10	Chloroplast	5	Cytoplasm	87	Nucleus	12	2	Cytosol	Li et al., 2010
Flavin-monooxygenase glucosinolate S-oxygenase 5	AT1G12140	FMOGS-OX5	A8MRX0	459	Chloroplast	Undefined	Mitochondrion	8	Chloroplast	5	Cytoplasm	87	Nucleus	12	2	Cytosol	Li et al., 2010

Flavin-monooxygenase glucosinolate S-oxygenase 6	AT1G12130	AtFMOGS-OX6	Q9FWW3	459	Chloroplast	Undefined	Plastid	12	Mitochondrion	4	Cytoplasm	87	Chloroplast	12	2		
Flavin-monooxygenase glucosinolate S-oxygenase 7	AT1G12160	AtFMOGS-OX7	Q9FWW6	470	Chloroplast	Undefined	Mitochondrion	10	Chloroplast	5	Cytoplasm	89	Plasma membrane	11	2		
Alkenyl hydroxalkyl producing 1	AT4G03070	AtAOP1	Q9ZTA3	322	Cytoplasm	Undefined	Cytosol	10	Any other	3	Cytoplasm	80	Nucleus	18	2		
Alkenyl hydroxalkyl producing 2	AT4G03060	AOP2		399	Cytoplasm	Undefined	Undefined		Undefined		Cytoplasm	88	Plasma Membrane	13	2	Cytoplasm	Zhang et al., 2015
2-oxoglutarate-dependent dioxygenase	AT4G03050	AOP3	Q9ZTA1	361	Cytoplasm	Secreted	Golgi, Cytosol	12	Secretory	5	Cytoplasm	83	Chloroplast	16	2	Cytosol	Jensen et al., 2015
2-Oxoglutarate-dependent dioxygenase	AT2G25450	GS-OH	Q9SKK4	359	Cytoplasm	Other (e.g., Cytoplasm)	Cytosol	22	Any other	2	Cytoplasm	87	Cytoplasm	16	3	Cytoplasm	
Myb transcription factor 28	AT5G61420	MYB28/HAG1/PMG1	Q9SPG2	366	Nucleus	Undefined	Nucleus	48	Any other	2	Nucleus	99	Nucleus	37	4	Nucleus	Yatusevich 2008
Myb transcription factor 29	AT5G07690	MYB29/PMG2/RAO7	Q9FLR1	366	Nucleus	Other (e.g., Cytoplasm)	Nucleus	30	Any other	2	Nucleus	93	Nucleus	35	4	Nucleus	Yatusevich 2008
Myb transcription factor 76	AT5G07700	MYB76	Q9SPG5	338	Nucleus	Other	Nucleus	30	Any other	2	Nucleus	92	Nucleus	35	4	Nucleus	Yatusevich 2008
**INDOLIC GLUCOSINOLATE BIOSYNTHESIS AND MODIFICATION**																	
Tryptophan synthase beta 1	AT5G54810	TSB1	P14671	470	Chloroplast	Chloroplast	Plastid	70	Chloroplast	1	Cytoplasm	88	Nucleus	14	3	Chloroplast	
Anthranilate synthase, alpha subunit, component I-1	AT5G05730	ASA1	P32068	624	Chloroplast	Undefined	Plastid	68	Chloroplast	5	Cytoplasm	81	Nucleus	13	2		
Cytochrome P450 79A2	AT5G05260	CYP79A2	Q9FLC8	523	ER	Undefined	ER	18	Mitochondrion	5	ER membrane	85	ER	14	4	ER	
Cytochrome P450 79B2	AT4G39950	CYP79B2	O81346	541	ER	Undefined	Cytosol	12	Any other	3	ER membrane	86	ER	15	3	ER	
Cytochrome P450 79B3	AT2G22330	CYP79B3	Q501D8	543	ER	Undefined	Plasma membrane	10	Any other	4	ER membrane	85	Nucleus	13	2		
Cytochrome P450 83B1	AT4G31500	CYP83B1	O65782	499	ER	Undefined	ER	56	Secretory	5	ER	83	ER	13	4	ER	
Glutathione S-transferase F9	AT2G30860	GSTF9	O80852	215	Cytoplasm	Undefined	Cytosol	32	Any other	4	Cytoplasm	95	Cytoplasm	60	3	Cytoplasm	
Glutathione S-transferase F10	AT2G30870	GSTF10	P42761	215	Cytoplasm	Undefined	Cytosol	38	Secretory	5	Cytoplasm	89	Cytoplasm	38	3	Cytoplasm	
UDP-glucosyl transferase 74B1	AT1G24100	UGT74B1	O48676	460	Cell membrane, Chloroplast.	Undefined	Plasma membrane	14	Any other	3	Cytoplasm	84	Chloroplast	12	2		
Sulfotransferase 5a	AT1G74100	AtSOT16/ST5a	Q9C9D0	338	Cytoplasm	Other (e.g., Cytoplasm)	Cytosol	38	Any other	2	Cytoplasm	99	Cytoplasm	84	3	Cytoplasm	Klein et al., 2006
Cytochrome P450 81F2	AT5G57220	CYP81F2	Q9LVD6	491	ER	Secreted	ER	28	Secretory	2	ER	82	Plasma membrane	13	3	ER	Fuchs et al., 2016
Cytochrome P450 81F4	AT4G37410	CYP81F4	Q9SZU1	501	ER	Undefined	ER	54	Secretory	4	ER membrane	82	ER	13	4	ER	
Myb transcription factor 51	AT1G18570	MYB51	O49782	352	Nucleus	Undefined	Nucleus	28	Any other	2	Nucleus	91	Nucleus	42	4	Nucleus	Gigolashvili et al., 2007
Myb transcription factor 34	AT5G60890	MYB34	O64399	295	Nucleus	Other (e.g., Cytoplasm)	Nucleus	44	Any other	2	Nucleus	90	Nucleus	44	4	Nucleus	
Myb transcription factor 122	AT1G74080	MYB122	Q9C9C8	333	Nucleus	Undefined	Nucleus	30	Any other	3	Nucleus	91	Nucleus	35	4	Nucleus	
Gamma-glutamyl peptidase 1	AT4G30530	GGP1	Q9M0A7	250	Chloroplast	Secreted	Cytosol	20	Secretory	5	Cytoplasm	82	Cytoplasm	20	2	Cytosol	Geu-Flores et al., 2011
Supper root 1	AT2G20610	SUR1	Q9SIV0	462	Chloroplast	Undefined	Cytosol	14	Any other	3	Mitochondrion	80	Nucleus	15	0		
Indole glucosinolate O-methyltransferase 1	AT1G21100	AtIGMT1	Q9LPU5	373	Chloroplast	Other (e.g., Cytoplasm)	Cytosol	24	Any other	1	Cytoplasm	82	Nucleus	14	2		
Indole glucosinolate O-methyltransferase 2	AT1G21120	AtIGMT2	Q9LPU7	373	Chloroplast	Other (e.g., Cytoplasm)	Cytosol	24	Any other	1	Cytoplasm	82	Cytoplasm	13	2	Cytoplasm	
Indole glucosinolate O-methyltransferase 3	AT1G21110	AtIGMT3	Q9LPU6	373	Chloroplast	Other (e.g., Cytoplasm)	Cytosol	22	Any other	1	Cytoplasm	82	Cytoplasm	13	2	Cytoplasm	
Indole glucosinolate O-methyltransferase 4	AT1G21130	AtIGMT4	Q3ED84	373	Chloroplast	Other (e.g., Cytoplasm)	Cytosol	24	Any other	1	Cytoplasm	83	Cytoplasm	14	2	Cytoplasm	
Indole glucosinolate O-methyltransferase 5	AT1G76790	AtIGMT5	Q9SRD4	367	Chloroplast	Other (e.g., Cytoplasm)	Cytosol	24	Any other	2	Cytoplasm	83	Plasma membrane	14	2		
**BENZOYLOXY GLUCOSINOLATEBIOSYNTHESIS**																	
Myb transcription factor 115	AT5G40360	MYB115	Q1PDP9	359	Nucleus	Undefined	Nucleus	28	Any other	4	Nucleus	87	Mitochondria	13	3	Nucleus	Wang et al., 2017
Myb transcription factor 118	AT3G27785	MYB118	Q9LVW4	437	Nucleus	Undefined	Nucleus	48	Any other	5	Nucleus	86	Nucleus	18	4	Nucleus	Du et al., 2018
**GLUCOSINOLATE TRANSPORT**																	
Nitrate transporter 2.10/ Glucosinolate transporter 1	AT3G47960	AtNPF2.10/GTR1	Q944G5	636	Vacuole	Undefined	Plasma membrane	36	Any other	5	Plasma membrane	87	Plasma membrane	75	3	Plasma membrane	Nour-Eldin et al., 2012
Nitrate transporter 2.11/ Glucosinolate transporter 2	AT5G62680	AtNPF2.11/GTR2	Q9LV10	616	Vacuole	Other (e.g. Cytoplasm)	Plasma membrane	32	Any other	1	Plasma membrane	87	Plasma membrane	56	3	Plasma membrane	Nour-Eldin et al., 2012
Nitrate transporter 1.9	AT1G18880	NPF2.9/NRT1.9	Q9M9V7	587	Vacuole	Other (e.g. Cytoplasm)	Plasma membrane	14	Any other	2	Plasma membrane	86	Plasma membrane	38	3	Plasma membrane	Wang and Tsay, 2011

Nitrate transporter 1.7	AT1G69870	NRT1.7	Q8RX77	620	Vacuole	Undefined	Plasma membrane	34	Any other	2	Plasma membrane	99	Plasma membrane	78	3	Plasma membrane	Fan et al., 2009
Nitrate transporter 1.6	AT1G27080	NRT1.6	Q9LFX9	576	Vacuole	Other (e.g. Cytoplasm)	Plasma membrane	44	Any other	1	Plasma membrane	99	Plasma membrane	76	3	Plasma membrane	Almagro et al., 2008
**MYROSINASE**																	
Atypical myrosinase PYK10	AT3G09260	PYK10	Q9SR37	524	ER	Secreted	ER	62	Secretory	1	ER	99	ER	73	4	ER	Matsushima et al., 2003
Syntaxin	AT3G11820	PEN1	Q9ZSD4	346	Golgi apparatus	Undefined	Plasma membrane	60	Any other	4	Golgi membrane	81	Nucleus	21	2	Plasma membrane	Collins et al., 2003; Assaad et al., 2004
Atypical myrosinase PEN2	AT2G44490	PEN2/BGLU26	O64883	560	Vacuole	Undefined	peroxisomes	52	Mitochondrion	4	peroxisome	99	Peroxisome	70	3	Peroxisomes, Mitocondria	Lipka et al., 2005; Fuchs et al., 2016
AtPDR8 ABC transporter	AT1G59870	PEN3	Q9XIE2	1469	Cell membrane, Chloroplast	Chloroplast	Plasma membrane	68	Chloroplast	3	Plasma membrane	99	Plasma membrane	76	4	Plasma membrane	Kobae et al., 2006; Stein et al., 2006
Phytochelatin synthase 1	AT5G44070	PCS1	Q9S7Z3	485	Nucleus	Undefined	Cytosol	36	Any other	3	Nucleus	87	Nucleus	13	3	Cytoplasm	Blum et al., 2010
Thioglucoside glucohydrolase 1	AT5G26000	TGG1/BGLU38	P37702	541	Vacuole	Secreted	Vacuole	46	Secretory	1	Vacuole	94	Vacuole	64	4	Vacuole	Andreasson et al., 2001; Ueda et al., 2006
Thioglucoside glucohydrolase 2	AT5G25980	TGG2/BGLU37	Q9C5C2	547	Vacuole	Secreted	Extracellular, Golgi, Vacuole	26	Secretory	1	Vacuole	94	Vacuole	59	4	Vacuole	Andreasson et al., 2001; Ueda et al., 2006
Thioglucoside glucohydrolase 3	AT5G48375	TGG3/BGLU39	Q3E8E5	439	Vacuole	Secreted	ER	18	Secretory	2	Vacuole	92	Vacuole	51	3	Vacuole	
Thioglucoside glucohydrolase 4	AT1G47600	TGG4/BGLU34	Q8GRX1	511	Vacuole	Secreted	Extracellular	30	Secretory	1	Vacuole	88	Chloroplast	21	2		
Thioglucoside glucohydrolase 5	AT1G51470	TGG5/BGLU35	Q3ECS3	511	Vacuole	Secreted	Extracellular	32	Secretory	1	Vacuole	88	Chloroplast	22	2		
Thioglucoside glucohydrolase 6	AT1G51490	TGG6	Q9C8K1	484	Vacuole	Mitochondrion	Mitochondrion	18	Mitochondrion	2	Vacuole	86	Chloroplast	24	3	Mitochondrion	
**MYROSINASE BINDING PROTEIN (MBP) AND MYROSINASE ASSOCICIATED PROTEINS (MyAP)**																	
Myrosinase-binding protein (MBP)	AT1G52040	MBP1	Q9SAV0	462	Cell wall, Cytoplasm, Mitochondrion	Other (e.g. Cytoplasm)	Nucleus	24	Any other	1	Cytoplasm	88	Nucleus	13	2		
Myrosinase-binding protein	AT1G52030	MBP2	Q9SAV1	642	Nucleus	Other (e.g. Cytoplasm)	Cytosol	12	Any other	2	Cytoplasm	87	Nucleus	19	2		
Myrosinase-binding protein	AT3G16450	JAL33	O04311	300	Cell wall, Chloroplast, Mitochondrion	Other (e.g. Cytoplasm)	Nucleus	34	Any other	2	Cytoplasm	90	Chloroplast	17	2		
Epithiospecifier modifier 1	AT3G14210	ESM1		392	Extracellular	Secreted	Extracellular	44	Secretory	1	Secreted	81	Cytoplasm	14	3	Secreted	
GDSL-like Lipase/Acylhydrolase superfamily protein	AT3G14220	GDSL-like Lipase	Q9LJP2	363	Extracellular	Secreted	Extracellular	40	Secretory	1	Secreted	81	Extracellular or Secreted	12	4	Extracellular	
Modified Vacuole Phenotype1	AT1G54030	MVP1	Q7XA74	417	Cell membrane, Extracellular	Undefined	Vacuole	42	Any other	3	Secreted	83	Extracellular or Secreted	13	2	ER	Agee et al., 2010
GDSL-like Lipase	AT1G54020	GLIP	Q9C5N8	372	Extracellular	Undefined	Vacuole	30	Secretory	1	Secreted		Nucleus	12	2		
GLL23	AT1G54010	GLL23	Q8W4H8	386	Extracellular	Secreted	Extracellular	44	Secretory	1	Secreted	81	Chloroplast	12	3	Secreted	
GLL22	AT1G54000	GLL22	Q1H583	391	Cell membrane, Extracellular	Secreted	Extracellular	44	Secretory	1	Secreted	81	Chloroplast	18	3	Secreted	
Vacuolar Morphology3/ Syntaxin of plants 22	AT5G46860	VAM3/SYP22	P93654	268	Vacuole	Undefined	Vacuole	64	Mitochondrion	5	ER	81	Vacuole	63	3	vacuolar membrane	Uemura et al., 2010
**SPECIFIER PROTEINS**																	
Nitrile specifier protein 1	AT3G16400	AtNSP1	Q9SDM9	470	Nucleus	Other (e.g. Cytoplasm)	Mitochondrion	14	Any other	2	Cytoplasm	88	Cytoplasm	16	2		
Nitrile specifier protein 2	AT2G33070	AtNSP2	O49326	473	Nucleus	Other (e.g. Cytoplasm)	Cytosol	12	Any other	2	Cytoplasm	89	Nucleus	17	2		
Nitrile specifier protein 3	AT3G16390	AtNSP3	O04318	467	Nucleus	Other (e.g. Cytoplasm)	Plastid	20	Any other	2	Cytoplasm	88	Cytoplasm	15	2		
Nitrile specifier protein 4	AT3G16410	AtNSP4	O04316	619	Nucleus	Other (e.g. Cytoplasm)	Extracellular , Cytosol	12	Any other	2	Cytoplasm	90	Nucleus	13	2		
Nitrile specifier protein 5	AT5G48180	AtNSP5	Q93XW5	326	Nucleus	Undefined	Cytosol	22	Any other	4	Nucleus	80	Nucleus	19	3	Nucleus	
Nitrile specifier protein 6	AT3G07720	AtNSP6	Q9S7W4	329	Nucleus	Undefined	Nucleus	36	Any other	5	Cytoplasm	80	Nucleus	23	3	Nucleus	
Epithiospecifier protein	AT1G54040	AtESP	Q8RY71	341	Nucleus	Undefined	Nucleus	46	Any other	5	Cytoplasm	80	Nucleus, Cytoplasm	45	3	Cytosol	Burow et al., 2007
Harmless to ozone layer1	AT2G43910	AtHOL1	Q0WP12	246	Chloroplast. Cytoplasm	Other (e.g. Cytoplasm)	Cytosol	36	Any other	2	Cytoplasm	82	Chloroplast	23	2	Cytoplasm	
**OTHER RELATED PROTEINS**																	
Like heterochromatin protein	AT5G17690	LHP1/TFL2	Q946J8	445	Nucleus	Undefined	Nucleus	70	Any other	2	Nucleus	99	Nucleus	67	4	Nucleus	Zemach et al., 2006

Dof-type zinc finger DNA-binding family protein	AT1G07640	OBP2/At DOF1.1	Q8L9V6	339	Nucleus	Undefined	Nucleus	64	Chloroplast	5	Nucleus	93	Nucleus	28	4	Nucleus	Yanagisawa, 2001
Basic helix-loop-helix (BHLH) DNA-binding superfamily protein	AT3G24140	FAMA/FMA	Q56YJ8	414	Nucleus	Other (e.g. Cytoplasm)	Nucleus	68	Any other	2	Nucleus	99	Nucleus	77	4	Nucleus	Shirakawa et al., 2014
Sulfur limitation 1/Ethylene-insensitive3-like	AT1G73730	SLIM1	O23116	567	Nucleus	Other (e.g. Cytoplasm)	Nucleus	48	Any other	2	Nucleus	92	Nucleus	31	4	Nucleus	Maruyama-Nakashita et al., 2006
LHP1-interacting factor 2	AT4G00830	Lif2	Q9ASP6	495	Chloroplast	Other (e.g. Cytoplasm)	Nucleus	14	Any other	2	Mitochondrion	82	Nucleus	19	2	Nucleus, Cytoplasm	Latrasse et al., 2011
Tetratricopeptide repeat (TPR)-like superfamily protein	AT1G04770	SDI2	Q9MAS6	303	Nucleus	Undefined	Mitochondrion	10	Mitochondrion	5	Cytoplasm	88	Nucleus	15	2		
Sulfur deficiency-induced 1	AT5G48850	ATSDI1	Q9FKB2	306	Nucleus	Other (e.g. Cytoplasm)	Nucleus	12	Any other	2	Cytoplasm	89	Chloroplast	18	2	Nucleus	Aarabi et al., 2016
Benzoyloxyglucosinolate 1	AT1G65880	BZO1	Q9SS01	580	Peroxisome	Undefined	peroxisomes	36	Any other	2	peroxisome	86	Plasma membrane	12	3	peroxisome	Quan et al., 2013
Mitogen-activated kinase 3	AT3G45640	AtMPK3	Q39023	370	Nucleus	Other (e.g. Cytoplasm)	Cytosol	34	Any other	2	Nucleus	91	Nucleus	35	3	Nucleus	Guan et al., 2014
Mitogen-activated kinase 6	AT2G43790	AtMPK6	Q39026	395	Nucleus	Other (e.g. Cytoplasm)	Cytosol	42	Any other	2	Nucleus	92	Nucleus	37	3	Nucleus	Guan et al., 2014
Ethylene response factor 6	AT4G17490	AtERF6	Q8VZ91	282	Nucleus	Undefined	Nucleus	48	Any other	4	Nucleus	93	Nucleus	36	4	Nucleus, Cytoplasm	Wang et al., 2013
BHLH transcription factors	AT1G32640	MYC2	Q39204	282	Nucleus	Undefined	Nucleus	40	Any other	2	Nucleus	83	Nucleus	27	4	Nucleus	Chini et al., 2009
BHLH transcription factors	AT5G46760	MYC3	Q9FIP9	592	Nucleus	Undefined	Nucleus	40	Any other	4	Nucleus	83	Nucleus	26	4	Nucleus	Cheng et al., 2011; Fernández-Calvo et al., 2011
BHLH transcription factors	AT4G17880	MYC4	O49687	589	Nucleus	Other (e.g. Cytoplasm)	Nucleus	40	Any other	2	Nucleus	84	Nucleus	29	4	Nucleus	Fernández-Calvo et al., 2011
BHLH transcription factors	AT5G46830	MYC5	Q9LUK7	511	Nucleus	Undefined	Nucleus	48	Any other	3	Nucleus	82	Nucleus	19	4	Nucleus	Qi et al., 2015
WRKY DNA-binding protein 18	AT4G31800	WRKY18	Q2V3D1	310	Nucleus	Undefined	Nucleus	60	Any other	4	Nucleus	84	Nucleus	25	4	Nucleus	Geilen and Böhmer, 2015
WRKY DNA-binding protein 40	AT1G80840	WRKY40	Q9SAH7	302	Nucleus	Undefined	Nucleus	58	Any other	2	Nucleus	86	Nucleus	24	4	Nucleus	Geilen and Böhmer, 2015
**NITRILASE**																	
Nitrilase 1	AT3G44310	AtNIT1	P32961	346	Cell membrane	Undefined	Plastid	32	Any other	5	Cytoplasm	82	Plasma membrane	80	2		
Nitrilase 2	AT3G44300	AtNIT2	P32962	339	Cell membrane	Undefined	Plasma membrane	22	Any other	4	Cytoplasm	82	Plasma membrane	84	3	Plasma membrane	Bartling et al .,1994 ; Grsic-Rausch et al., 2000
Nitrilase 3	AT3G44320	AtNIT3	P46010	346	Cell membrane	Undefined	Plastid	16	Any other	4	Cytoplasm	81	Plasma membrane	85	2		
Nitrilase 4	AT5G22300	AtNIT4	P46011	355	Cell membrane	Undefined	Plasma membrane	14	Any other	3	Cytoplasm	80	Plasma membrane	85	3		
**SULFUR METABOLISM**																	
Adenosine-5'-phosphosulfate kinase 2	AT4G39940	AKN2/APK2	O49196	293	Chloroplast	Undefined	Plastid	52	Chloroplast	4	Chloroplast	99	Chloroplast	47	5	Plastid, Chloroplast	Mugford et al., 2009
Adenosine-5'-phosphosulfate kinase 1	AT2G14750	APK1	Q43295	276	Chloroplast	Chloroplast	Plastid	64	Chloroplast	3	Chloroplast	99	Chloroplast	77	6	Chloroplast	Mugford et al., 2009
**RuBisCO**	AT5G14260	RuBisCO	Q8VZB5	541	Chloroplast	Chloroplast	Plastid	58	Chloroplast	1	Chloroplast	99	Chloroplast	12	6	Chloroplast	Jensen and Bassham, 1968

References:

1. Aarabi, F., Kusajima, M., Tohge, T., Konishi, T., Gigolashvili, T., Takamune, M., et al. (2016). Sulfur deficiency–induced repressor proteins optimize glucosinolate biosynthesis in plants. *Science Advances*, *2*(10), e1601087.
2. Agee, A. E., Surpin, M., Sohn, E. J., Girke, T., Rosado, A., Kram, B. W., et al. (2010). MODIFIED VACUOLE PHENOTYPE1 is an Arabidopsis myrosinase-associated protein involved in endomembrane protein trafficking. *Plant Physiology*, *152*(1), 120-132.
3. Almagro, A., Lin, S. H., & Tsay, Y. F. (2008). Characterization of the Arabidopsis nitrate transporter NRT1. 6 reveals a role of nitrate in early embryo development. *The Plant Cell*, *20*(12), 3289-3299.
4. Andréasson, E., Jørgensen, L. B., Höglund, A. S., Rask, L., & Meijer, J. (2001). Different myrosinase and idioblast distribution in Arabidopsis and *Brassica napus*. *Plant Physiology*, *127*(4), 1750-1763.
5. Assaad, F. F., Qiu, J. L., Youngs, H., Ehrhardt, D., Zimmerli, L., Kalde, M., et al. (2004). The PEN1 syntaxin defines a novel cellular compartment upon fungal attack and is required for the timely assembly of papillae. *Molecular Biology of the Cell*, *15*(11), 5118-5129.
6. Bartling, D., Seedorf, M., Schmidt, R. C., & Weiler, E. W. (1994). Molecular characterization of two cloned nitrilases from *Arabidopsis thaliana*: key enzymes in biosynthesis of the plant hormone indole-3-acetic acid. *Proceedings of the National Academy of Sciences*, *91*(13), 6021-6025.
7. Blum, R., Meyer, K. C., Wünschmann, J., Lendzian, K. J., & Grill, E. (2010). Cytosolic action of phytochelatin synthase. *Plant Physiology*, *153*(1), 159-169.
8. Burow, M., Rice, M., Hause, B., Gershenzon, J., & Wittstock, U. (2007). Cell-and tissue-specific localization and regulation of the epithiospecifier protein in *Arabidopsis thaliana*. *Plant Molecular Biology*, *64*(1-2), 173-185.
9. Cheng, Z., Sun, L., Qi, T., Zhang, B., Peng, W., Liu, Y., & Xie, D. (2011). The bHLH transcription factor MYC3 interacts with the jasmonate ZIM-domain proteins to mediate jasmonate response in Arabidopsis. *Molecular plant*, *4*(2), 279-288.
10. Chini, A., Fonseca, S., Chico, J. M., Fernández- Calvo, P., & Solano, R. (2009). The ZIM domain mediates homo- and heteromeric interactions between Arabidopsis JAZ proteins. *The Plant Journal*, *59*(1), 77-87.
11. Collins, N. C., Thordal-Christensen, H., Lipka, V., Bau, S., Kombrink, E., Qiu, J. L., et al. (2003). SNARE-protein-mediated disease resistance at the plant cell wall. *Nature*, *425*(6961), 973-977.
12. Diebold, R., Schuster, J., Däschner, K., & Binder, S. (2002). The branched-chain amino acid transaminase gene family in Arabidopsis encodes plastid and mitochondrial proteins. *Plant Physiology*, *129*(2), 540-550.
13. Du, Y. T., Zhao, M. J., Wang, C. T., Gao, Y., Wang, Y. X., Liu, Y. W., et al. (2018). Identification and characterization of GmMYB118 responses to drought and salt stress. *BMC Plant Biology*, *18*(1), 320.
14. Fan, S. C., Lin, C. S., Hsu, P. K., Lin, S. H., & Tsay, Y. F. (2009). The Arabidopsis nitrate transporter NRT1.7, expressed in phloem, is responsible for source-to-sink remobilization of nitrate. *The Plant Cell*, *21*(9), 2750-2761.

15. Fernández-Calvo, P., Chini, A., Fernández-Barbero, G., Chico, J. M., Gimenez-Ibanez, S., Geerinck, J., et al. (2011). The Arabidopsis bHLH transcription factors MYC3 and MYC4 are targets of JAZ repressors and act additively with MYC2 in the activation of jasmonate responses. *The Plant Cell*, *23*(2), 701-715.
16. Fuchs, R., Kopischke, M., Klapprodt, C., Hause, G., Meyer, A. J., Schwarzländer, M., et al. (2016). Immobilized subpopulations of leaf epidermal mitochondria mediate PENETRATION2-dependent pathogen entry control in Arabidopsis. *The Plant Cell*, *28*(1), 130-145.
17. Geilen, K., & Böhmer, M. (2015). Dynamic subnuclear relocalisation of WRKY40 in response to abscisic acid in *Arabidopsis thaliana*. *Scientific reports*, *5*, 13369.
18. Geu-Flores, F., Møldrup, M. E., Böttcher, C., Olsen, C. E., Scheel, D., & Halkier, B. A. (2011). Cytosolic γ-glutamyl peptidases process glutathione conjugates in the biosynthesis of glucosinolates and camalexin in Arabidopsis. *The Plant Cell*, *23*(6):2456-2469..
19. Gigolashvili, T., Yatusevich, R., Berger, B., Müller, C., & Flügge, U. I. (2007). The R2R3-MYB transcription factor HAG1/MYB28 is a regulator of methionine-derived glucosinolate biosynthesis in *Arabidopsis thaliana*. *The Plant Journal*, *51*(2), 247-261.
20. Grsic-Rausch, S., Kobelt, P., Siemens, J. M., Bischoff, M., & Ludwig-Müller, J. (2000). Expression and localization of nitrilase during symptom development of the clubroot disease in Arabidopsis. *Plant Physiology*, *122*(2), 369-378.
21. Guan, Y., Lu, J., Xu, J., McClure, B., & Zhang, S. (2014). Two mitogen-activated protein kinases, MPK3 and MPK6, are required for funicular guidance of pollen tubes in Arabidopsis. *Plant Physiology*, *165*(2), 528-533.
22. He, Y., Chen, B., Pang, Q., Strul, J. M., & Chen, S. (2010). Functional specification of Arabidopsis isopropylmalate isomerases in glucosinolate and leucine biosynthesis. *Plant and Cell Physiology*, *51*(9), 1480-1487.
23. He, Y., Mawhinney, T. P., Preuss, M. L., Schroeder, A. C., Chen, B., Abraham, L., et al. (2009). A redox-active isopropylmalate dehydrogenase functions in the biosynthesis of glucosinolates and leucine in Arabidopsis. *The Plant Journal*, *60*(4), 679-690.
24. Jensen, L. M., Kliebenstein, D. J., & Burow, M. (2015). Investigation of the multifunctional gene AOP3 expands the regulatory network fine-tuning glucosinolate production in Arabidopsis. *Frontiers in Plant Science*, *6*, 719.
25. Jensen, R. G., & Bassham, J. A. (1968). Photosynthesis by isolated chloroplasts III. Light activation of the carboxylation reaction. *Biochimica et Biophysica Acta (BBA)-Bioenergetics*, *153*(1), 227-234.
26. Klein, M., Reichelt, M., Gershenzon, J., & Papenbrock, J. (2006). The three desulfoglucosinolate sulfotransferase proteins in Arabidopsis have different substrate specificities and are differentially expressed. *The FEBS Journal*, *273*(1), 122-136.
27. Knill, T., Reichelt, M., Paetz, C., Gershenzon, J., & Binder, S. (2009). Arabidopsis thaliana encodes a bacterial-type heterodimeric isopropylmalate isomerase involved in both Leu biosynthesis and the Met chain elongation pathway of glucosinolate formation. *Plant Molecular Biology*, *71*(3), 227-239.
28. Kobae, Y., Sekino, T., Yoshioka, H., Nakagawa, T., Martinoia, E., & Maeshima, M. (2006). Loss of AtPDR8, a Plasma membrane ABC transporter of *Arabidopsis thaliana*, causes hypersensitive cell death upon pathogen infection. *Plant and Cell Physiology*, *47*(3), 309-318.

29. Kroymann, J., Textor, S., Tokuhisa, J. G., Falk, K. L., Bartram, S., Gershenzon, J., & Mitchell-Olds, T. (2001). A gene controlling variation in Arabidopsis glucosinolate composition is part of the methionine chain elongation pathway. *Plant Physiology*, *127*(3), 1077-1088.
30. Latrasse, D., Germann, S., Houba-Hérin, N., Dubois, E., Bui-Prodhomme, D., Hourcade, D., et al. (2011). Control of flowering and cell fate by LIF2, an RNA binding partner of the polycomb complex component LHP1. *PloS One*, *6*(1), e16592.
31. Li, J., Kristiansen, K. A., Hansen, B. G., & Halkier, B. A. (2010). Cellular and subcellular localization of flavin-monooxygenases involved in glucosinolate biosynthesis. *Journal of Experimental Botany*, *62*(3), 1337-1346.
32. Lipka, V., Dittgen, J., Bednarek, P., Bhat, R., Wiermer, M., Stein, M., et al. (2005). Pre-and postinvasion defenses both contribute to nonhost resistance in Arabidopsis. *Science*, *310*(5751), 1180-1183.
33. Maruyama-Nakashita, A., Nakamura, Y., Tohge, T., Saito, K., & Takahashi, H. (2006). Arabidopsis SLIM1 is a central transcriptional regulator of plant sulfur response and metabolism. *The Plant Cell*, *18*(11), 3235-3251.
34. Matsushima, R., Kondo, M., Nishimura, M., & Hara-Nishimura, I. (2003). A novel ER-derived compartment, the ER body, selectively accumulates a β-glucosidase with an ER-retention signal in Arabidopsis. *The Plant Journal*, *33*(3), 493-502.
35. Mugford, S. G., Yoshimoto, N., Reichelt, M., Wirtz, M., Hill, L., Mugford, S. T., et al. (2009). Disruption of adenosine-5′-phosphosulfate kinase in Arabidopsis reduces levels of sulfated secondary metabolites. *The Plant Cell*, *21*(3), 910-927.
36. Nintemann, S. J., Vik, D., Svozil, J., Bak, M., Baerenfaller, K., Burow, M., & Halkier, B. A. (2017). Unravelling protein-protein interaction networks linked to aliphatic and indole glucosinolate biosynthetic pathways in Arabidopsis. *Frontiers in Plant Science*, *8*, 2028.
37. Nour-Eldin, H. H., Andersen, T. G., Burow, M., Madsen, S. R., Jørgensen, M. E., Olsen, C. E., et al. (2012). NRT/PTR transporters are essential for translocation of glucosinolate defence compounds to seeds. *Nature*, *488*(7412), 531-534.
38. Qi, T., Huang, H., Song, S., & Xie, D. (2015). Regulation of jasmonate-mediated stamen development and seed production by a bHLH-MYB complex in Arabidopsis. *The Plant Cell*, *27*(6), 1620-1633.
39. Quan, S., Yang, P., Cassin-Ross, G., Kaur, N., Switzenberg, R., Aung, K., et al. (2013). Proteome analysis of peroxisomes from etiolated Arabidopsis seedlings identifies a peroxisomal protease involved in β-oxidation and development. *Plant Physiology*, *163*(4), 1518-1538.
40. Reintanz, B., Lehnen, M., Reichelt, M., Gershenzon, J., Kowalczyk, M., Sandberg, G., et al. (2001). bus, a bushy Arabidopsis CYP79F1 knockout mutant with abolished synthesis of short-chain aliphatic glucosinolates. *The Plant Cell*, *13*(2), 351-367.
41. Schuster, J., Knill, T., Reichelt, M., Gershenzon, J., & Binder, S. (2006). Branched-chain aminotransferase4 is part of the chain elongation pathway in the biosynthesis of methionine-derived glucosinolates in Arabidopsis. *The Plant Cell*, *18*(10), 2664-2679.
42. Shirakawa, M., Ueda, H., Nagano, A. J., Shimada, T., Kohchi, T., & Hara-Nishimura, I. (2014). FAMA is an essential component for the differentiation of two distinct cell types, myrosin cells and guard cells, in Arabidopsis. *The Plant Cell*, *26*(10), 4039-4052.
43. Stein, M., Dittgen, J., Sánchez-Rodríguez, C., Hou, B. H., Molina, A., Schulze-Lefert, P., et al. (2006). Arabidopsis PEN3/PDR8, an ATP binding cassette transporter, contributes to

nonhost resistance to inappropriate pathogens that enter by direct penetration. *The Plant Cell*, *18*(3), 731-746.
44. Textor, S., De Kraker, J. W., Hause, B., Gershenzon, J., & Tokuhisa, J. G. (2007). MAM3 catalyzes the formation of all aliphatic glucosinolate chain lengths in Arabidopsis. *Plant Physiology*, *144*(1), 60-71.
45. Ueda, H., Nishiyama, C., Shimada, T., Koumoto, Y., Hayashi, Y., Kondo, M., et al. (2006). AtVAM3 is required for normal specification of idioblasts, myrosin cells. *Plant and Cell Physiology, 47*(1), 164-175.
46. Uemura, T., Morita, M. T., Ebine, K., Okatani, Y., Yano, D., Saito, C., et al. (2010). Vacuolar/pre-vacuolar compartment QaSNAREs VAM3/SYP22 and PEP12/SYP21 have interchangeable functions in Arabidopsis. *The Plant Journal*, *64*(5), 864-873.
47. Wang, L., Ran, L., Hou, Y., Tian, Q., Li, C., Liu, R., et al. (2017). The transcription factor MYB115 contributes to the regulation of proanthocyanidin biosynthesis and enhances fungal resistance in poplar. *New Phytologist*, *215*(1), 351-367.
48. Wang, P., Du, Y., Zhao, X., Miao, Y., & Song, C. P. (2013). The MPK6-ERF6-ROSE7/GCC-box complex modulates oxidative gene transcription and the oxidative response in *Arabidopsis thaliana*. *Plant physiology*, *161*(3):1392-1408.
49. Wang, Y. Y., & Tsay, Y. F. (2011). Arabidopsis nitrate transporter NRT1. 9 is important in phloem nitrate transport. *The Plant Cell*, *23*(5), 1945-1957.
50. Yanagisawa, S. (2001). The transcriptional activation domain of the plant-specific Dof1 factor functions in plant, animal, and yeast cells. *Plant and Cell Physiology*, *42*(8), 813-822.
51. Yatusevich, R. (2008). Analysis of the MYB28, MYB29 and MYB76 transcription factors involved in the biosynthesis of aliphatic glucosinolates in *Arabidopsis thaliana*. *Doctoral dissertation*, Universität zu Köln, Germany.
52. Zemach, A., Li, Y., Ben-Meir, H., Oliva, M., Mosquna, A., Kiss, V., et al. (2006). Different domains control the localization and mobility of LIKE HETEROCHROMATIN PROTEIN1 in Arabidopsis nuclei. *The Plant Cell*, *18*(1), 133-145.
53. Zhang, J., Liu, Z., Liang, J., Wu, J., Cheng, F., & Wang, X. (2015). Three genes encoding AOP2, a protein involved in aliphatic glucosinolate biosynthesis, are differentially expressed in *Brassica rapa*. *Journal of Experimental Botany*, *66*(20), 6205-6218.
